# High *miRNA-378* expression has high diagnostic values for pulmonary tuberculosis and predicts adverse outcomes

**DOI:** 10.1186/s12860-022-00413-w

**Published:** 2022-03-19

**Authors:** Xiaolu Sun, Kai Liu, Yan Zhao, Tianhua Zhang

**Affiliations:** 1 Department of Tuberculosis Treatmen and Prevention, Shaanxi Provincial Institute for Tuberculosis Control and Prevention, 4# of Xingqing South Road, Xi’an, 710048 Shaanxi China; 2 Department of Orthopedics, First Affiliated Hospital of Xi’an Jiaotong University, Xi’an, China

**Keywords:** *miR-378*, Pulmonary tuberculosis, Drug-resistant TB, Immunological function, Adverse outcomes, Receiver operating characteristic curve, Pearson coefficient, Diagnostic value

## Abstract

**Background:**

Pulmonary tuberculosis (TB) is a chronic infectious disease. microRNA (miR)-378 is involved in TB diagnosis. This study explored the effects of *miR-378* on TB patients.

**Methods:**

A total of 126 TB patients were selected, including 63 active TB and 63 latent TB, with 62 healthy subjects as controls. Serum *miR-378* expression was detected. The diagnostic value of *miR-378* in TB was analyzed using the ROC curve. Immune inflammatory factor levels were detected and their correlations with *miR-378* expression were analyzed. The drug resistance of active TB patients was recorded after standard treatment. *miR-378* expression in drug-resistant TB patients was detected. The effects of *miR-378* on adverse outcome incidence were analyzed.

**Results:**

*miR-378* expression was highly expressed in TB and the expression was higher in the active group than the latent group. Serum miR-378 expression > 1.490 had high sensitivity and specificity in TB diagnosis. *miR-378* expression was correlated with TB clinical indexes. IL-4, IL-6, and IL-1β levels were highly expressed, while IFN-γ, TNF-α, and IL-12 levels were lowly expressed in TB patients. Serum *miR-378* level in the active group was positively correlated with serum IL-4, IL-6, and IL-1β, and negatively correlated with serum IFN-γ, TNF-α, and IL-12 concentrations. *miR-378* expression was downregulated in the TB treated, single (SDR TB) and multi-drug resistance (MDR TB) groups, the *miR-378* expression in SDR TB and MDR TB groups was higher than the TB treated group and lower in the SDR TB group than the MDR TB group. High *miR-378* expression predicted higher adverse outcome incidence.

**Conclusions:**

High *miR-378* expression assisted TB diagnosis and predicted adverse outcomes.

## Introduction

Pulmonary tuberculosis (TB) is a chronic contagious infectious disease, which is transmitted almost exclusively through cough aerosol, caused by the Mycobacterium tuberculosis complex, and characterized by the necrotizing granulomatous inflammation in the lungs, although almost any of the extra-pulmonary site can be affected [1]. Currently, the drug-resistant forms of TB are tending to be the most lethal pathogen worldwide, responsible for a quarter of deaths due to drug resistance [2]. The risk factors including the socioeconomic position of the family, housing in fewer windows, and indoor air pollution, are powerful predictors of the infection and disease of TB, and people who have suffered from TB once have an increased risk of developing TB again, further exacerbating the vicious cycle of TB and poverty [3]. The procedure of the clinical diagnosis for TB is time-consuming and inadequate, often leading to delayed diagnosis or misdiagnosis, thereby increasing the incidence rate and mortality or overtreatment risks [4]. Due to the evolution of Mycobacterium tuberculosis strains, the infection stage varies from latent period to drug resistance, and as a result, the disease is complex. In general one-third of patients showed latent infection, since Mycobacterium tuberculosis is in a latent state for a long time, and is prone to active diseases in the case of low immune function [5]. This latent disease is a threat to humans. Therefore, a strategy to identify Mycobacterium tuberculosis antigens with diagnostic potential is to identify genes specifically induced during infection or at a specific disease stage. The disadvantages of current immunodiagnosis include the inability to detect the progression from latent infection to active TB, and the inability to monitor the therapeutic effect [6]. In addition, the need for a sputum-independent POC test has been highlighted by the World Health Organization (WHO) in their high-priority target product [7], which highlights the need for new TB biomarkers. These biomarkers should be highly sensitive and specific in the diagnosis of TB infection, and can clearly distinguish latent and active TB.

Several serological proteins for the diagnosis of TB have been reported [8, 9]. However, poor stability and complex operation limit the repeatability of protein detection [10], which further limits their clinical application. microRNAs (miRNAs) are a class of small, non-coding RNAs with a length of 21–23 nt and regulate the expression of genes at the post-translational level, bind to sites in 3’ untranslated region (3’UTR) of target genes, and lead to the degradation of translational repression or transcript, which are well known for the diverse functions in biological processes, such as cell differentiation, proliferation, death, and apoptosis, especially in TB infection [11]. Serum miRNAs are easier and more stable to detect and as a result, serum miRNAs are potentially good biomarkers for many diseases [12]. Recent research has identified miRNAs as candidate biomarkers for TB [5, 13, 14]. Various serum miRNAs including miR-378 show significant differences among TB patients and healthy people [10]. miR-378 was preliminarily determined to be one of the specific miRNAs in the serum of patients with TB [10], but the relationship between miR-378, latent tuberculosis infection (LTBI), and active TB has not been reported.

The occurrence of TB will lead to changes in the immune system [15]. The immune response to TB infection mainly depends on CD4 + T cells [16]. CD4 + T lymphocytes can differentiate into a variety of effector cells such as Th1, Th2, Th17, and regulatory T cells (Treg). The imbalance between Th1/Th2 and Th2 is of great significance in the peripheral immune response in the development of TB [17]. Th1 cells mainly produce IFN-γ, TNF-α, and IL-12 to control intracellular infection including mycobacterium tuberculosis, while Th2 cells produce IL-4, IL-5, IL-6, IL-13, and IL-1β to mediate fluid immunization [18]. In patients with Mycobacterium tuberculosis infection, the immune response usually differentiates into Th2 cells as a form of escape [19]. IFN-γ/IL-4 can be used as an effective serum biomarker combination for LTBI and active TB screening [20]. miR-378 has been reported to be involved in regulating the immune process of tumors, muscular atrophy, atherosclerosis, and other diseases [21–23]. During the infection of Mycobacterium tuberculosis, miR-378d functions on decreasing Mycobacterium tuberculosis intracellular survival [24]. Therefore, we hypothesized that the expression of miR-378 was related to the immune system and could play a role in assisting the diagnosis of TB. The purpose of this study was to explore the relationship between serum miR-378 and the clinical case parameters of patients with TB and its diagnostic value.

## Materials and methods

### Ethics statement

The experiments were authorized by the academic ethics committee of Shaanxi Provincial Institute for Tuberculosis Control and Prevention (2,020,072,212). All procedures were strictly implemented by the code of ethics. All subjects involved were fully informed of the objective of the study and signed informed consent before sampling.

### Study subjects

This study prospectively included 126 patients with TB (study group) hospitalized in Shaanxi Provincial Institute for Tuberculosis Control and Prevention from June 2018 to May 2020. The patients were assigned to 2 subgroups according to their conditions including 63 cases of active TB (active group) and 63 cases of latent TB infection (latent group) detected by physical examination. In addition, 62 people who accepted physical examination in Shaanxi Provincial Institute for Tuberculosis Control and Prevention in the same period were selected (control group). The whole blood samples were collected from all subjects (centrifuged at 1500 × g at 4 °C for 10 min and stored at -70 °C). The grouping was implemented according to the standard of Diagnosis for pulmonary tuberculosis in 2018 issued by the National Health and Family Planning Commission.

### Inclusion and exclusion criteria

Diagnostic criteria for active TB were as follows: typical clinical symptoms, chest X-ray and computed tomography images, and positive sputum culture for Mycobacterium tuberculosis.

Diagnostic criteria for latent TB were as follows: following the manufacturer’s guidelines, all participants were screened by QuantiFERON-TB (QFT) Gold test (Qiagen, France). Participants with positive results, normal chest X-ray results, and without clinical symptoms were considered to be latent TB.

The criteria for healthy subjects in the same period were as follows: negative QuantiFERON-TB test result, no immune diseases, excluded TB infection after examination, and complete clinical general data.

Exclusion criteria were as follows: drop out of the experiment, re-treated TB, complicated with malignant tumors, a history of immunopreparations in the past half of the year, and a history of anti-TB treatment before grouping, and lost visitors.

### Inclusion criteria of drug-resistant TB group

Among the 63 patients with active TB, 17 had not started anti-TB treatment (TB naïve group), and the remaining 46 patients were treated with standard treatment regimens, including rifampicin and isoniazid. Patients without significant improvement in clinical symptoms after treatment were acid-fast bacilli (AFB) positive at the end of 16 weeks and were tested for rifampicin and isoniazid mutations, and were classified as single drug-resistant (SDR) and multi-drug-resistant (MDR) TB. The specific grouping was as follows: incomplete anti-TB therapy group (TB naive group, *N* = 17), single drug-resistance group (SDR TB group, *N* = 12), multi-drug-resistance group (MDR TB group, *N* = 15), and no drug-resistance group (TB-treated group, *N* = 19).

### Data collection and follow-up

The data of all enrolled subjects when they were enrolled were recorded, including gender, age, weight, smoking, drinking, and TB classification of TB patients and normal subjects, and expectoration, chest pain, low fever, and hemoptysis in the active group and latent group. The patients with TB were followed up for 6 months and the adverse outcomes were documented. The adverse outcomes were defined as death, adverse reactions, follow-up failure, and turning to drug resistance. The death referred to the patients who died for any reason during the treatment. Adverse reactions referred to harmful or adverse reactions caused by drugs in patients during the treatment. Follow-up failure referred to the patients who interrupted treatment for 2 consecutive months or more. Turning to drug resistance referred to the resistance of patients to 1 or more anti-TB drugs after receiving treatment for a period of time [25, 26].

### Enzyme-linked immunosorbent assay (ELISA)

Immune and inflammatory factors were then detected. The levels of IFN-γ, IL-4 (I-bio, Fuzhou, Jiangxi, China, IB-E10033, IB-E10051), TNF-α, IL-6, IL-12 (RAB0478, RAB0306, RAB0252, Sigma-Aldrich, St. Louis, MO, USA), and IL-1β (ab9722, Abcam, Cambridge, UK) in the serum of miR-378 high and low expression groups were detected in strict accordance with the instructions of ELISA kits. The samples were incubated with the coating solution in the ELISA plates at 37 °C for 2 h and then sealed with 10% calf serum at 4 °C overnight. The samples were washed, incubated with primary antibody at 37 °C for 2 h, and incubated with secondary antibody at 37 °C for 1 h. After development, the samples were added with termination solution, and the optical density (OD) value of each well was measured at 450 nm.

### Total RNA extraction and reverse transcription quantitative polymerase chain reaction (RT-qPCR)

The total RNA was extracted using TRIzol reagent (ZCIBIO Technology Co., Ltd., Shanghai, China, ZC-0021A). The total RNA was transcribed into cDNA using PrimeScript RT reagent kits (Shrbio, Nanjing, Jiangsu, China, DRR037A). The qPCR was performed on the ABI7900HT fast PCR real-time system (Yihbio, Shanghai, China) using SYBR®Premix Ex Taq™ II (Shanranbio, Shanghai, China). The reaction conditions were as follows: pre-denaturation at 95 °C for 10 min and 40 cycles of denaturation at 95 °C for 10 s, annealing at 60 °C for 20 s and extending at 72 °C for 34 s. With U6 as the internal reference, the data were analyzed using the 2^−ΔΔCt^ method. The primers were synthesized by Shanghai Sangon Biotech and the sequences were shown in Table 1 [27].

**Table 1:** Real-time PCR primer sequence

Gene	Forward 5’-3’	Reverse 5’-3’
*miR-378*	5’-CTGAGACTGGACTTGGAGTC-3’	5’-GTGCAGGGTCCGAGGT-3’
U6	5’-TGCGGGTGCTCGCTTCGGCAGC-3’	5’-CCAGTGCAGGGTCCGAGGT-3’

### Data analysis

SPSS 21.0 statistical software (IBM Corp. Armonk, NY, USA) and GraphPad Prism 6.0 software (GraphPad Software Inc, San Diego, CA, USA) were used for data statistical analyses and mapping. The Shapiro–Wilk test was used to test the normal distribution of data. Counting data were expressed as cases/percentage (n/%) and compared using the Chi-square test. Measurement data were expressed as mean ± standard error of mean (SEM) and the data between two groups were compared using independent *t* test, and the data among groups were compared using One-way analysis of variance (ANOVA), followed by Tukey's multiple comparisons test. The value of miR-378 in differentiating TB was analyzed using the receiver operating characteristic (ROC) curve and the effect of miR-378 expression on the incidence of adverse outcomes was analyzed using the Chi-square test and Kaplan–Meier method. The Log rank method was used to test the differences between groups of Kaplan–Meier curve. *P* < 0.05 was indicative of statistical significance.

## Results

### Clinical baseline characteristics of the subjects

A total of 126 TB patients (including 63 cases of active TB and 63 cases of latent TB) were included, and 62 subjects accepted health examination in Shaanxi Provincial Institute for Tuberculosis Control and Prevention during the same period were selected as the controls. There were no significant differences in gender, age, weight, drinking history, and other clinical baseline data between the 3 groups (*P* > 0.05), there were significant differences in M. tuberculosis positive infection, chronic bronchitis, and chronic obstructive pulmonary disease (COPD) between the active group and the control group, and the clinical indexes such as smoking history, expectoration, chest pain, low fever, hemoptysis, and M. tuberculosis positive infection in the active group and the latent group were significantly different from those in the control group (all *P* < 0.05, Table 2).

**Table 2:** Comparison of general baseline data

factors	Control (*N* = 62)	Study (*N* = 126)	
-	-	Active (*N* = 63)	Latent (*N* = 63)
Gender (female/male)	(37/25)	(37/26)	(42/21)
Age (≤ 45/ > 45)	(24/38)	(27/36)	(28/35)
Weight (≤ 60/ > 60)	(28/34)	(26/37)	(30/33)
Smoking history (yes/no)	(45/17)	(39/24)^ab^	(42/21)^a^
Drinking history (yes/no)	(35/27)	(51/12)	(49/14)
Expectoration (yes/no)	-	(58/5)^ab^	(47/16)^a^
Chest pain (yes/no)	-	(59/4)^ab^	(43/20)^a^
Low fever (yes/no)	-	(54/9)^ab^	(42/21)^a^
Hemoptysis (yes/no)	-	(57/6)^ab^	(14/49)^a^
M. tuberculosis culture positivity (yes/no)	-	(52/11)^ab^	-
Chronic bronchitis (yes/no)	(7/55)	(19/44)^a^	(13/50)
COPD (yes/no)	(5/57)	(14/49)^a^	(7/56)
Diabetes (yes/no)	(8/54)	(11/52)	(7/56)
Hypertension (yes/no)	(8/54)	(15/48)	(7/56)
Hyperlipidemia (yes/no)	(9/53)	(15/48)	(13/50)

*Note*: The measurement data was expressed by the number of cases, and the comparison between the two groups was performed by independent *t*-test. *M. tuberculosis* Mycobacterium tuberculosis, *COPD* chronic obstructive pulmonary disease

^a^compared with the control group, *P* < 0.05

^b^compared with the latent group, *P* < 0.05

### *miR-378* was highly expressed in the serum of TB patients and had high diagnostic value

**Fig. 1:**
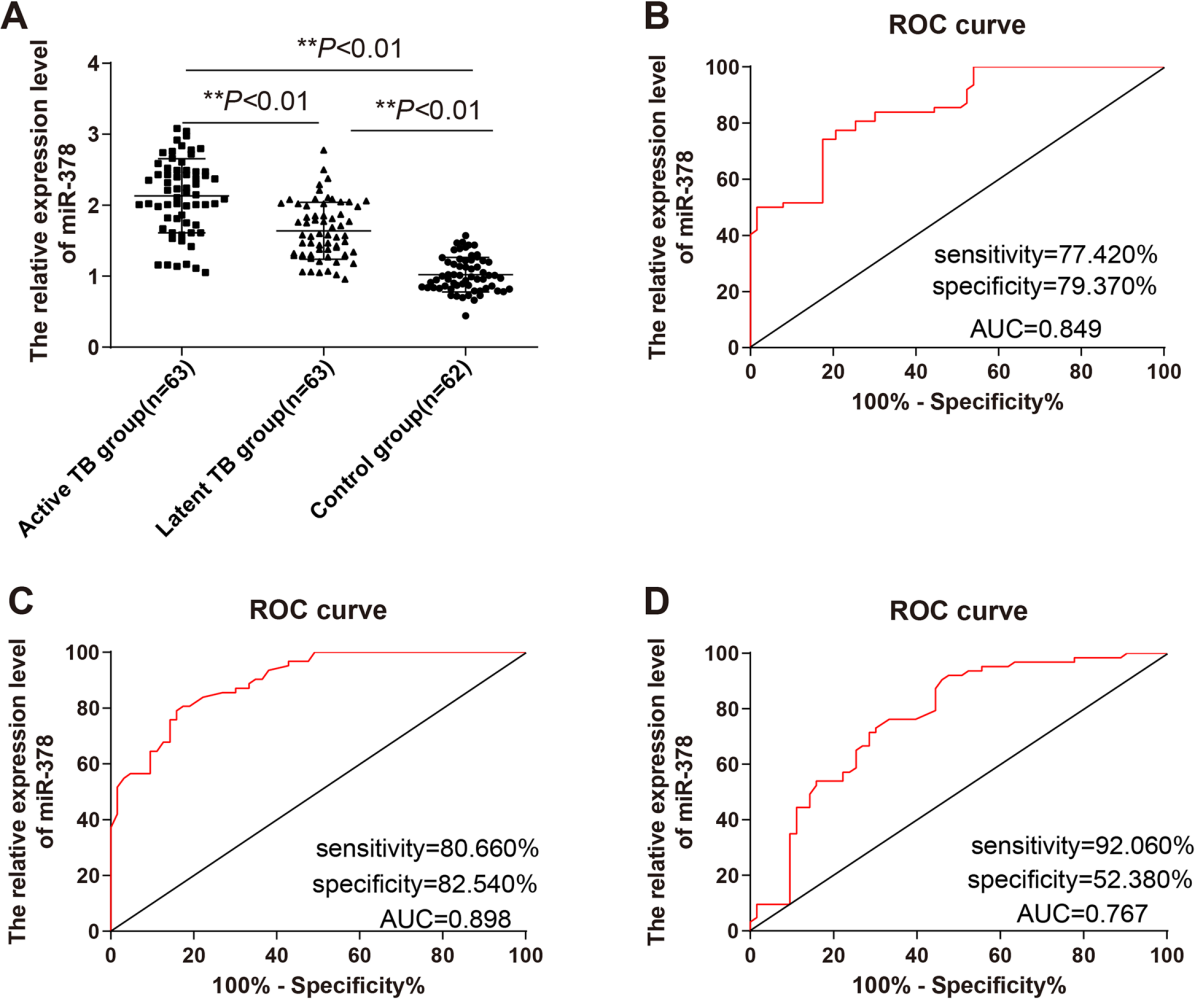
*miR-378* was highly expressed in the serum of TB patients and had high diagnostic value. **A** The expression of *miR-378* in the serum of TB patients in the active group and latent group and normal subjects was detected using RT-qPCR; **B** The diagnostic efficacy of *miR-378* in active TB was analyzed using the ROC curve; **C** The diagnostic efficacy of *miR-378* in latent TB was analyzed using the ROC curve; **D** The diagnostic efficacy of *miR-378* in active and latent TB was analyzed using the ROC curve. One-way ANOVA was used for data analysis in panel (**A)**, followed by Tukey's multiple comparisons test, and the ROC curve was used for data analysis in panels **B**, **C**, and **D**. ** *P* < 0.01. Active TB group (*N* = 63), latent TB group (*N* = 63), control group (*N* = 62). The expression of miR-378 in the control group was normalized to 1. The expression of miR-378 in other groups was relative to that of miR-378 in the control group

The expression of *miR-378* in the serum of normal subjects and TB patients in the active group and latent group was compared. Compared with the control group, *miR-378* was upregulated in the active group and latent group, and the *miR-378* expression in the active group was higher than the latent group (*P* < 0.05, Fig. 1A). Furthermore, the ROC curve of *miR-378* for TB diagnosis was drawn (Fig. 1B-D). The area under the curve (AUC) of *miR-378* for distinguishing the normal group and active group was 0.849, with the cut-off value of 1.490 (the sensitivity was 77.420% and the specificity was 79.370%) (Fig. 1B); AUC of *miR-378* for distinguishing the normal group and latent group was 0.898, with the cut-off value of 1.275 (the sensitivity was 80.660% and the specificity was 82.540%) (Fig. 1C). The AUC of *miR-378* for distinguishing the active group and the latent group was 0.767, with the cut-off value of 2.130 (the sensitivity was 92.060% and the specificity was 52.380%) (Fig. 1D). These results suggested that serum expression of *miR-378* < 1.490 could assist the diagnosis of TB.

### Correlation of *miR-378* and clinical baseline characteristics of TB patients

The correlation of *miR-378* and clinical baseline characteristics of TB patients was further studied. The expression of *miR-378* had no significant correlation with gender, age, weight, and drinking history of TB patients (all *P* > 0.05), while had significant correlations with smoking history, expectoration, chest pain, low fever, and hemoptysis (all *P* < 0.05, Table 3).

**Table 3:** miR-378 expression was independently correlated with the activity of TB patients

	Logistics univariate regression analysis			Logistics multivariate regression analysis		
	*P*	OR	95% CI	*P*	OR	95% CI
Gender	0.474	1.294	0.640–2.616	/	/	/
Age	0.857	1.067	0.527–2.157	/	/	/
Weight	0.474	1.294	0.640–2.616	/	/	/
Smoking history	0.577	0.813	0.392–1.686	/	/	/
Drinking history	0.66	1.214	1.851–9.160	/	/	/
Expectoration	0.012	3.949	1.347–11.574	0.482	2.826	0.156–51.273
Chest pain	0.001	6.86	2.187–21.520	0.984/	0.967	0.037–25.562
Low fever	0.014	3	1.246–7.224	0.553	2.786	0.095–82.072
Hemoptysis	0	33.25	11.873–93.114	0.022	33.497	1.666–673.456
Chronic bronchitis	0.222	1.661	1.021–1.092	/	/	/
COPD	0.066	2.5	0.942–6.638	0.883	0.69	0.005–96.796
IFN-γ	0	0.933	0.908–0.959	0.697	1.02	0.922–1.129
IL-4	0	1.242	1.154–1.336	0.018	1.274	1.042–1.558
TNF-α	0.344	0.44	0.080–2.408	/	/	/
IL-6	0	1.139	1.089–1.191	0.012	1.182	1.038–1.347
IL-12	0	0.845	0.794–0.899	0.015	0.754	0.600–0.947
IL-1β	0.17	2.878	0.636–13.032	/	/	/
Diabetes	0.312	1.692	0.610–4.693	/	/	/
Hypertension	0.066	2.5	0.942–6.638	0.728	0.519	0.013–21.007
Hyperlipidemia	0.668	1.202	0.518–2.789	/	/	/
miR-378	0	8.791	3.621–21.340	0.047	39.016	1.054–1444.199

### miR-378 expression was independently correlated with the activity of TB patients

**Fig. 2:**
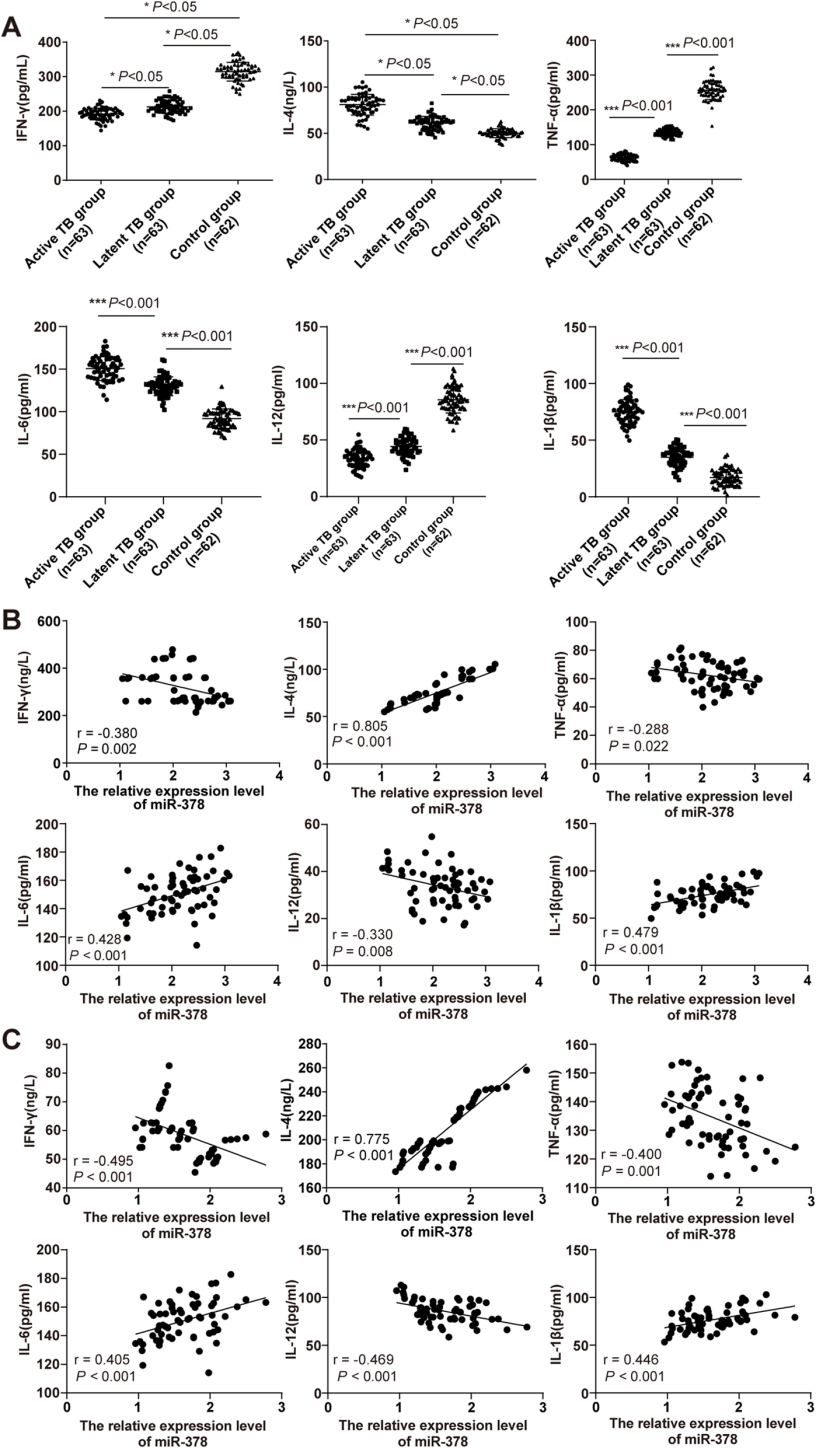
Correlation between *miR-378* expression and immune function in TB patients. **A** The levels of IFN-γ, IL-4, TNF-α, IL-6, IL-12, and IL-1β in TB patients in the active group and latent group and normal subjects were detected using ELISA; Pearson analysis method analyzed the correlation between the expression of miR-378 and (**B**) IFN-γ, IL-4, TNF-α, IL-6, IL-12, and   IL-1β in the serum of patients in the active group and the correlation between the expression of miR-378 and (**C**) IFN-γ, IL-4, TNF-α, IL-6, IL-12, and IL-1β in the serum of patients in the latent groups. One-way ANOVA was used for data analysis in panel (**A**), followed by Tukey's multiple comparisons test. * *P* < 0.05, *** *P* < 0.001. Pearson coefficient analysis was used for data analysis in panels **B** and **C**. Active TB group (*N* = 63), latent TB group (*N* = 63), control group (*N* = 62)

TB is a chronic infectious disease, which is related to patient’s autoimmune function. In recent years, due to the specific expression of inflammatory factors, immune function, and other related indicators in TB patients, it has become a hot spot in clinical research [28]. miR-378, as a posttranscriptional regulatory factor, has been playing an important role in regulating immune cells [29]. The levels of immune-inflammatory factors IFN-γ, IL-4, TNF-α, IL-6, IL-12, and IL-1β in the control group, active group, and latent group were observed. Compared with the control group, IFN-γ, TNF-α, and IL-12 levels were decreased and IL-4, IL-6, and IL-1β levels were increased in the active group and the latent group, while compared with the latent group, IFN-γ, TNF-α, and IL-12 levels were decreased and IL-4, IL-6, and IL-1β levels were increased in the active group (all *P* < 0.05, Fig. 2A). To study the relationship between miR-378 and IFN-γ, IL-4, TNF-α, IL-6, IL-12, and IL-1β in TB patients, the Pearson method was applied for detection. Serum miR-378 level in the active group and the latent group was negatively correlated with serum IFN-γ, TNF-α, and IL-12 concentrations, while positively correlated with IL-4, IL-6, and IL-1β concentrations (Fig. 2B-C).

The independent correlation between miR-378 expression and clinicopathological characteristics of TB patients was further studied. First, the clinical indexes and activity of TB patients were detected by Logistics univariate regression analysis. Then, active TB was taken as the dependent variable, and according to the results of univariate regression analysis, the clinical indexes such as expectoration, chest pain, low fever, hemoptysis, COPD, IFN-γ, IL-4, IL-6, IL-12, hypertension, and miR-378 with *P* < 0.1 were included as independent variables in the multivariate logistic regression analysis. After adjusting expectoration, chest pain, low fever, COPD, IFN-γ, hypertension, hemoptysis, IL-4, IL-6, and IL-12, miR-378 was independently correlated with the activity of TB patients (*P* = 0.047, OR = 39.016, 95% CI: 1.054–1444.199) (Table 3).

### *miR-378* was highly expressed in the serum of patients with MDR TB

**Fig. 3:**
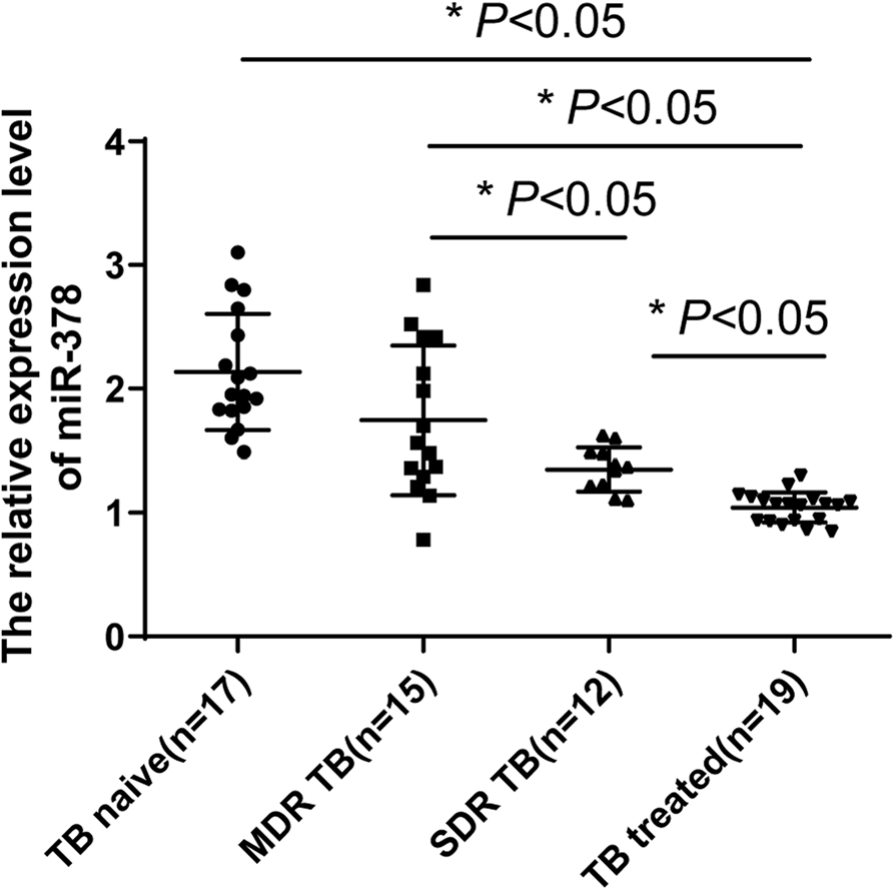
miR-378 was highly expressed in the serum of patients with drug-resistant TB. The expression changes of *miR-378* in the serum of patients in the TB naive group, TB treated group, SDR TB group, and MDR TB group were detected using RT-qPCR. One-way ANOVA was used for data analysis, followed by Tukey's multiple comparisons test. * *P* < 0.05. TB naive group (incomplete antinuclear therapy, *N* = 17), SDR TB group (single drug resistance group, *N* = 12), MDR TB group (multidrug resistance group, *N* = 15), TB treated group (non-drug resistance group, *N* = 19)

The 46 patients in the active group had accepted anti-TB treatment (≥ 6 months), including 19 patients who had no drug resistance (TB treated group), and 27 patients who had multidrug resistance or single-drug resistance. The 27 patients were all confirmed as single drug-resistant (SDR TB group, *N* = 12) or MDR TB (MDR TB group, *N* = 15) by liner probe assay. To determine the association between *miR-378* and drug-resistant TB patients, the *miR-378* expression in the serum of TB naive group, TB treated group, SDR TB group, and MDR TB group was compared. Compared with the TB naive group, the *miR-378* was downregulated in the TB treated group, SDR TB group, and MDR TB group. Compared with the TB treated group, the *miR-378* was upregulated in the SDR TB group and MDR TB group and compared with the MDR TB group, the *miR-378* expression was downregulated in the SDR TB group (*P* < 0.05, Fig. 3). The results above suggested that *miR-378* was highly expressed in drug-resistant TB.

### High expression of *miR-378* predicted poor prognosis in TB patients

**Fig. 4:**
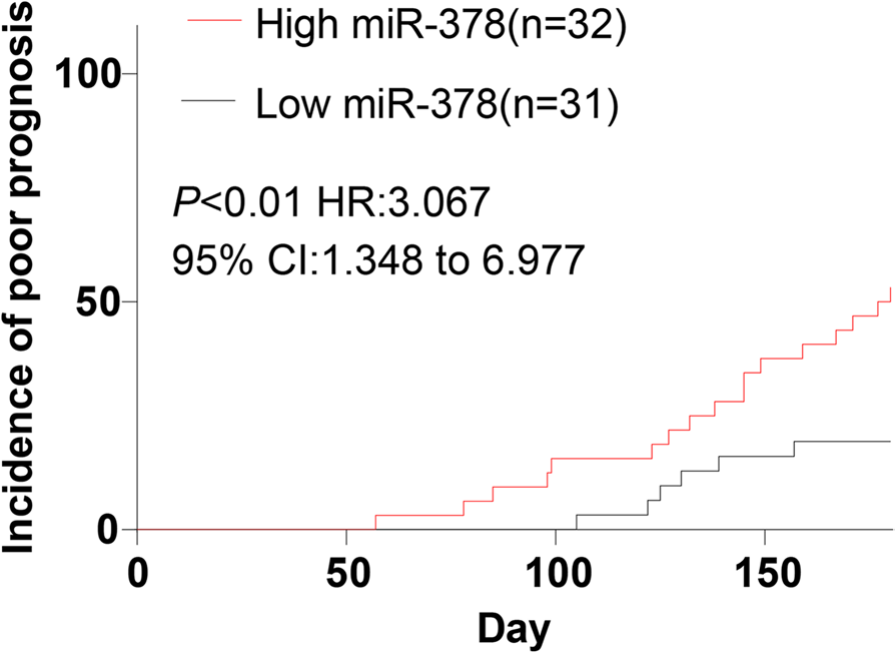
High expression of *miR-378* predicted poor prognosis in TB patients. The effect of *miR-378* expression on the incidence of adverse prognosis in TB patients within 6 months of treatment was analyzed using the Kaplan–Meier curve. The curve of the *miR-378* high expression group shifted to the left compared with the low expression group. Log-rank analysis was used to test the differences of Kaplan–Meier curve in different groups. High miR-378 (miR-378 high expression group, *N* = 32), low-miR-378 (miR-378 high expression group, *N* = 31)

In this study, the TB patients in the active group were assigned to low expression group (*N* = 31) and high expression group (*N* = 32) with the median expression of miR-378 as the boundary, and the incidence of the adverse prognosis within 6 months after treatment in the active group was predicted. There were differences in prognosis between the 2 groups (*P* < 0.05). The incidence of adverse outcomes in the low expression group was 6 cases (19.355%), which was lower than that in the high expression group (17 cases, 53.125%). Kaplan–Meier analysis demonstrated that the curve of the *miR-378* high expression group shifted to the left (*P* < 0.01, Fig. 4), indicating higher cumulative incidences of adverse outcomes in the high expression group. These results suggested that elevated *miR-378* expression was associated with a poor prognosis.

## Discussion

TB is a leading cause of death all over the world from infectious diseases among adults, which has been identified as an emergency for global public health for decades [3]. Evidence has shown the essential role of *miR-378*in TB [10]. This study illustrated that high expression of *miR-378* could assist the diagnosis of TB and predict adverse outcomes.

Various miRNAs have been identified as potential early biomarkers for TB [11, 30]. However, the expression and diagnostic value of *miR-378* in TB remained unclear. Our results demonstrated that compared with normal subjects, the *miR-378* was upregulated in the serum of active TB patients and latent TB patients and the *miR-378* expression in the active group was higher than the latent group. Consistently, *miR-378* was upregulated in TB patients [31]. To further study the expression of *miR-378* in the clinical diagnosis of TB, we plotted the ROC curve. Our results showed that the AUC of *miR-378* for distinguishing the normal group and the active group was 0.849 and the cut-off value was 1.490 with 77.420% sensitivity and 79.370% specificity; the AUC of *miR-378* for distinguishing the normal group and latent group was 0.898 and the cut-off value was 1.275 with 80.660% sensitivity and 82.540% specificity; the AUC of *miR-378* for distinguishing the active group and the latent group was 0.767 and the cut-off value was 2.130 with 92.060% sensitivity and 52.380% specificity. A recent study suggested that *miR-378* could be explored as a promising biomarker for TB [10]. In brief, serum *miR-378* < 1.490 could assist the diagnosis of TB and accurately differentiate latent TB from active TB. Furthermore, we studied the correlation between *miR-378* expression and clinicopathological features of patients with TB. Smoking and drinking are the risk factors of TB [32]. Symptoms of pulmonary tuberculosis are cough (100%), chest pain (80%), expectoration (100%), hemoptysis (33.3%), fever (93.33%), anorexia (86.66%), and loss of weight (80%) [33]. Our results demonstrated that the *miR-378* expression had significant correlations with smoking history, expectoration, chest pain, low fever, and hemoptysis.

The TB patients have the specific expression of inflammatory factors and immune function [28]. Indirect evidence supports the possibility that Mycobacterium tuberculosis induces a small but important subversive Th2 response in the dominant Th1 environment, leading to bacterial proliferation and disease progression [34]. IFN-γ is an immune factor and IL-4 is an inflammatory factor [35, 36]. Pro-inflammatory cytokines are signaling molecules produced by T cells, macrophages, and other immune cells, which can promote inflammation and immunity. Mainly based on animal model research, the cytokines, such as IFN-γ, IL-4, TNF-a, IL-6, IL-12, and IL-1β, are considered to play a major role in host resistance to TB [37]. IFN-γ level in active TB patients is diminished, while IL-4 level is increased; IFN-γ tends to increase during the transition from active TB to latent TB, while IL-4 tends to decrease during this process [18]. Our results revealed that compared with the normal subjects, IFN-γ, TNF-α, and IL-12 levels were decreased and IL-4, IL-6, and IL-1β levels were increased in the active TB patients and the latent TB patients, while compared with the latent TB patients, IFN-γ, TNF-α, and IL-12 levels were decreased and IL-4, IL-6, and IL-1β levels were increased in the active TB patients. Consistently, *miR-378* plays an essential role in immunopathogenesis as a posttranscriptional regulatory factor [29]. To study the relationship between *miR-378* and IFN-γ and IL-4 levels of TB patients, we performed the Person analysis. Our results showed that serum miR-378 level in the active group and the latent group was negatively correlated with serum IFN-γ, TNF-α, and IL-12 concentrations, while positively correlated with IL-4, IL-6, and IL-1β concentrations. Altogether, our results unraveled the association between serum miR-378 expression and the immune function of TB patients.

The management and control of TB worldwide are faced with the challenge of the worsening scenarios of drug resistance [38]. To determine the relationship between *miR-378* and drug-resistant TB patients, we compared the serum *miR-378* expression after drug treatment. Our results revealed that compared with TB naive patients, *miR-378* expression was reduced in TB treated patients, SDR TB patients, and MDR TB patients. Compared with TB-treated patients, *miR-378* expression was elevated in the SDR TB patients and MDR TB patients; and compared with MDR TB patients, the *miR-378* expression was reduced in the SDR TB patients. However, there is no report at present on the expression of *miR-378* in drug-resistant TB. This study found that *miR-378* was highly expressed in drug-resistant TB for the first time.

Furthermore, a variety of miRNAs are closely related to adverse outcomes of TB [39]. We analyzed the incidence of adverse outcomes of TB patients with the high and low expression of *miR-378*. Our results found that the adverse outcome incidence in patients with low *miR-378* expression was 6 cases (19.355%), which was lower than that of the patients with high *miR-378* expression (17 cases) (53.125%). The curve of the *miR-378* high expression group shifted to the left, indicating that the adverse outcome cumulative incidence was higher in patients with high *miR-378* expression. In brief, elevated *miR-378* expression was associated with a poor prognosis. The research of miRNA-mediated tuberculosis regulation is enormous. As a regulator and regulator of the response, it may regulate the disease progression through the influence of epigenetic mechanism (histone acetylation or DNA methylation) and its targeting pathway, which need further exploration.

In summary, as a prospective study, the expression of *miR-378* in the serum of TB patients was measured for the first time. The role of *miR-378* expression in the diagnosis of TB was investigated using the ROC curve. The effect of *miR-378* expression on the adverse outcomes of TB was determined using survival analysis and logistic regression analysis, which provided a new starting point for clinical condition judgment and adverse outcome prediction. However, the number of cases and events included in this study was less, so it is necessary to further expand the sample size and carry out multi-center research. The mechanism of *miR-378* in TB cells wasn’t discussed. In addition, due to the limitation of the experimental cycle and funds, we did not detect the changes in Th1/Th2 immune cell balance. These deficiencies need to be supplemented in future studies to further clarify the diagnostic and prognostic evaluation ability of *miR-378*. Further work is warranted to measure the expression of serum *miR-378* in TB cells and drug-resistant TB cells to study its effects on cell proliferation, apoptosis, and cell cycle.

## Data Availability

All the data generated or analyzed during this study are included in this published article.
